# Inversion of the Womb

**Published:** 1861

**Authors:** R. C. Hamill


### Selections.

### INVERSION OF THE WOMB.

CASE REPORTED TO THE CHICAGO ACADEMY OF MED. SCIENCES, FEB. 1ST, 1861.

BY R. C. HAMILL, M. D.

We copy the following case from the *Chicago Medical Journal*, in connection with the foregoing extract from the proceedings of the New York State Medical Society, chiefly because it so completely refutes the views of Dr. Lee, in some respects, and sustains those fully set forth by us, in the January number of the Examiner.—[Ed.]

Mrs. C——, an accomplished and intellectual lady, in her 26th year, was taken in labor with her first child, about 12 o'clock at night, Dec. 17th, 1860. She had suffered during her entire term of pregnancy from a highly exalted state of the brain and nervous system, amounting on many occasions to absolute mania. I found her perfectly calm when I was called in at 7 o'clock the following morning,—her pulse a little accelerated—80 per minute. The pains were good, occuring once in five or six minutes. The os uteri was dilated to the size of a dime, yielding readily at each pain. Her bowels had been freely evacuated just before my entrance. There was nothing in her situation to mark her case as differing from an ordinary labor under favorable circumstances, except, that I thought the antero-posterior diameter of the pelvis was slightly below the average dimensions.

There was nothing that required my immediate presence, and I left the house until half-past nine. On my return the head was found fairly engaged in the superior strait—the vertex toward the left acetabulum. At 11 o'clock the head began to press upon the perineum, which was remarkably thick and unyielding, and carried the head back promptly as the uterine contraction subsided. This state of the labor continued for more than an hour, when, after a long continued expulsive effort, she complained of pain in the head, and immediately began to talk incoherently, repeating scraps of poetry, sing songs and hymns, which were uninterrupted by the most vigorous pains. She had laid upon her left side, bearing all her pains in that position up to this time, but she was now uncontrollable, throwing herself about, from side to side, straightening herself, generally during the severity of the pain.

As early as ten o'clock she had desired me to give her chloroform. I declined, assuring her that she could bear her child without it—that there was nothing in her condition *then* calling for its effects, remarking that I always administered it with great anxiety, and only when I was convinced its aid was absolutely required. Under the present aspect of the case, and at the urgent wish of her husband, I concluded to give it, and accordingly ordered five or six drops on a clean handkerchief to be applied to her nose. The effect was speedily apparent, and the second portion, which was given at the next pain, produced quiet, which was kept up till the termination of the labor—from five to ten drops being given at the accession of nearly every pain. The child was born at 1 o'clock P. M.—a boy—weighing about eight pounds. On learning that it was a boy, she clapped her hands in delight and expressed the greatest anxiety to see it.

I waited, contrary to my usual custom, as long as ten minutes for the voluntary expulsion of the placenta. No uterine contraction occurring, I placed my left hand upon the abdomen and commenced gentle manipulation. In a few moments the womb contracted into a firm globular tumor, when I made traction upon the cord bringing the placenta down, so that my finger could readily reach the point from which the cord took its departure At this point it was held. Fearing that I would break the cord, which was small and weak, I ceased traction and applied my right hand over the uterine tumor, which was distinctly felt, of a regular globular shape, through the walls of the abdomen. My hand was not laid heavily upon it, neither did I make any pressure more than what was necessary to recognize its situation, when she cried out "Doctor, there is another child coming," and immediately began to strain violently. The uterus glided quickly into the pelvis, and she partially swooned. On carrying my hand to the vagina, which was done immediately, I found the placenta partly expelled and floating in a torrent of blood, about one-half of it still adherent to the inverted uterus. I peeled off the attached portion as quickly as possible, and grasping the inverted part, which was firmly contracted, and consisted of the body and fundus, endeavored its immediate reposition, but without success. I then tried to indent the fundus with my thumb, but could not make the slightest impression on it. Whilst the uterus was in my grasp I discovered that the walls of the left side appeared to be softer and thinner than any other portion of it, which fact suggested the idea of beginning the reposition at that point. Accordingly, with the fundus resting on the hollow of the hand, I made pressure with the ends of my fingers upon the weakest portion of the walls of the body of the womb, indenting it and at the same time pushing the fundus upward, I succeeded, with comparatively little effort, in carrying the inverted portion to its place, the fundus being the last to reach its normal position. My hand was not withdrawn until the contraction was complete. The patient rallied speedily and asked for her child. At this time Dr. Bevan, who had been sent for, arrived, and assisted in applying compress and bandage; for whose assistance then and subsequently, I bear the most grateful remembrance. I remained with her until three o'clock. She drank a cup of tea and I left her in as comfortable a condition as the nature of the case would justify me to expect.

At half-past four Dr. Bevan met me again at the bed-side. There was more waste of blood than was desirable; her pulse was feeble—116 per minute; the uterus, well contracted, was felt in its proper situation. She had "sunk away two or three times" for a moment, during my absence, and had a similar sinking after I came in. The color did not leave her lips, or the pulse flag during the time, which did not exceed a moment. At the suggestion of Dr. B., powders of the acetate of lead and opium were ordered, to be given once in two hours until the hemorrhage ceased. Four powders only were given during the night.

Dec. 19.—We saw her at 8 o'clock. She had a pretty comfortable night; slept some. About three pints of urine was drawn off, presenting nothing unusual in its character. Pulse 100. Effervescing drinks were ordered during the day; a little dry toast and lemonade allowed her. In the evening her pulse was 120, but fell to 100 after drawing off a pint of urine.

Her husband, whose solicitude was extreme, desired me to call in Dr. Byford, who saw her about 9 o'clock P. M., and expressed himself favorably impressed with her symptoms and condition. At 10 o'clock, five grs. Dover powder and the twelfth gr. morphine were given to procure rest, and another powder left to be given in four hours in case she does not sleep.

20th.—Both powders given; had some sleep and rested quietly; pulse 125; breasts a little swollen, with increased secretion of milk; lochia natural. When the child is put to the breast, complains of uterine contraction. No coagula. At each visit the fundus was felt over the pubes.

We saw her again at 6 in the evening. There had been some febrile excitement during the day which depended upon the secretion of milk. Her body and limbs were sponged with tincture of arnica, with evident relief to the muscular soreness and numbness. An emulsion of castor oil and turpentine, with an opiate was ordered at 8 P. M., to be repeated in the morning if her bowels were not moved.

21st.—Rested well, part of the night. Her bowels not moved; oil and turpentine mixture repeated, to be followed by an enema at 12 M. 6 P. M.—Bowels had been freely moved after the enema; passed urine without the aid of catheter; complained of burning pain in the urethra, which was considerably swollen; expressed herself as feeling very comfortable otherwise; pulse 100. Dr. Bevan discontinued his attention.

22d,—8 o'clock A. M.—Her mother and brother, who had been telegraphed from a distant city, arrived during the evening of yesterday, and her father late in the night. Their presence drew too largely upon her nervous system, and I found her this morning, to use her own words, "quite miserable." Had slept very little, and since 3 in the morning had suffered with pain in her back. Her tongue was slightly coated, but not dry; some headache; pulse 120; abundant secretion of milk; lochia natural in quality and quantity; free secretion of urine; the abdomen a little swollen, with some tenderness on pressure; a burning pain in the pelvis; no unusual heat of the vaginia, except along the course of the urethra, which was swollen and painful. I ordered calomel X grs., Dover's Powder XV grs., to be divided into three powders—one every two hours. A fine cloth moistened with a solution of morphine was laid upon the orifice of the urethra, and effervescing powders were ordered *ad libitum.*

Her husband, again growing alarmed, desired counsel and proposed Dr. Byford, who was sent for and met me at 12 M. Symptoms the same as in the morning; pulse increased to 128 per minute; complained of a burning pain deep in the pelvis. The opinion of Dr. B. was, that inflammation of the womb was to be feared, and that active medication to control such tendency was clearly indicated, and suggested that she should be put upon the use of veratrum viride immediately in full doses; accordingly verat. virid ʒ j, tinct. hyoscyamus ℥ ij was ordered—one teaspoonfull every six hours; and he further suggested that one grain of sulph. quinine should be given every six hours, alternating the two prescriptions; and ungt. belladonna was applied to the urethra. Turpentine and alcohol, had been applied to the abdomen in the morning and were continued.

23d.—Had rested better; very little pain anywhere; pulse 115. 12 M.—Met Dr. Byford at the bedside; pulse 110; general modification of all the symptoms; treatment continued, veratrum viride and quinia at increased intervals, once in eight hours each. Citrate of magnesia to move the bowels.

24th, 12 M.—We found her very comfortable, without pain anywhere; the bowels had been freely moved; pulse 80 and soft. Her case required little further treatment, and she is at this date, February 1st, 1861, enjoying uninterrupted health.

So much interest has been elicited on the subject of inversion of the uterus since the Fisher-Stone trial, that I have been induced to give a more minute history of this case and its attendant circumstances, than otherwise might have been deemed necessary. I have been practicing medicine for nearly 25 years, and have had a good proportion of obstetric services to perform during the greater portion of that time. This is the first and only case that has occurred in my hands, and it has induced me to modify my opinion in relation to the occurrence of that accident.

In this case the inversion could not have been prevented by any ordinary precaution—the eccentric state of the organ could not be discovered by any of the usual rules that govern the conduct of an accoucheur.

In the first place, we have an adhering placenta, large, filling the relaxed neck of the uterus; second, the walls of the body of the organ, weak and thin, at least on one side, with slight contraction: third, the fundus firmly contracted. With this condition of the parts, an expulsive effort could scarcely fail to carry the fundus through the unresisting body and neck, and eventuate in partial or complete inversion. Nothing but support from within, by the hand of the accoucheur, could have prevented, I am convinced, in this instance, the occurrence of this accident. Many of the cases attributed to unwarrantable traction upon the cord, and resulting to the serious prejudice of the attendant, may have depended upon the same, or a similar cause—the accoucheur as innocent of the result as the infant that has just been ushered into the world. The error consisting, not in the occurrence of the accident, but in the failure to recognize, and at the moment reposit the inverted organ.

